# Assessment of mobilization capacity in 10 different ICU scenarios by different professions

**DOI:** 10.1371/journal.pone.0239853

**Published:** 2020-10-15

**Authors:** Carsten Hermes, Peter Nydahl, Manfred Blobner, Rolf Dubb, Silke Filipovic, Arnold Kaltwasser, Bernhard Ulm, Stefan J. Schaller

**Affiliations:** 1 CCRN, Bonn, Germany; 2 Nursing Research, Department of Anesthesiology and Intensive Care Medicine, University Hospital of Schleswig-Holstein, Kiel, Germany; 3 Department of Anesthesiology and Intensive Care, Klinikum rechts der Isar, School of Medicine, Technical University of Munich, Munich, Germany; 4 Academy of District Clinics Reutlingen, Reutlingen, Germany; 5 Department of Physiotherapy, University Hospital of Giessen and Marburg, Marburg, Germany; 6 Academy of District Clinics Reutlingen, Reutlingen, Germany; 7 Corporate Member of Freie Universität Berlin, Charité –Universitätsmedizin Berlin, Berlin, Germany; 8 Humboldt-Universität zu Berlin, Charité –Universitätsmedizin Berlin, Berlin, Germany; 9 Department of Anesthesiology and Operative Intensive Care Medicine, Berlin Institute of Health, Berlin, Germany; Institute of Mental Health, SINGAPORE

## Abstract

**Background:**

Mobilization of intensive care patients is a multi-professional task. Aim of this study was to explore how different professions working at Intensive Care Units (ICU) estimate the mobility capacity using the ICU Mobility Score in 10 different scenarios.

**Methods:**

Ten fictitious patient-scenarios and guideline-related knowledge were assessed using an online survey. Critical care team members in German-speaking countries were invited to participate. All datasets including professional data and at least one scenario were analyzed. Kruskal Wallis test was used for the individual scenarios, while a linear mixed-model was used over all responses.

**Results:**

In total, 515 of 788 (65%) participants could be evaluated. Physicians (p = 0.001) and nurses (p = 0.002) selected a lower ICU Mobility Score (-0.7 95% CI -1.1 to -0.3 and -0.4 95% CI -0.7 to -0.2, respectively) than physical therapists, while other specialists did not (p = 0.81). Participants who classified themselves as experts or could define early mobilization in accordance to the “S2e guideline: positioning and early mobilisation in prophylaxis or therapy of pulmonary disorders” correctly selected higher mobilization levels (0.2 95% CI 0.0 to 0.4, p = 0.049 and 0.3 95% CI 0.1 to 0.5, p = 0.002, respectively).

**Conclusion:**

Different professions scored the mobilization capacity of patients differently, with nurses and physicians estimating significantly lower capacity than physical therapists. The exact knowledge of guidelines and recommendations, such as the definition of early mobilization, independently lead to a higher score. Interprofessional education, interprofessional rounds and mobilization activities could further enhance knowledge and practice of mobilization in the critical care team.

## Introduction

Early mobilization is defined according to the German S2e guideline “positioning and early mobilization in prophylaxis or therapy of pulmonary disorders” as mobilization within 72 hours after admission to an intensive care unit (ICU) [1]. Due to its proven benefits, such as shorter duration of mechanical ventilation [2], improved functionality [3], and possibly shorter duration of delirium [4–8], while being safe [9] and cost-effective [10], early mobilization is a highly recommended but hardly implemented therapy during intensive care.

Implementation of early mobilization is recommended in an inter-professional approach [5, 11–14]. In practice, professionals frequently report barriers to early mobilization, such as sedation, consciousness disorders, or hemodynamic or respiratory instability [15]. Interestingly, different professions report different barriers to early mobilization, e.g. nurses often report hemodynamic instability on continuous renal replacement therapy as barriers, while physical therapists report neurological impairments or inability to follow commands [16–24]. Hence, different professions may estimate the mobility of ICU patients differently.

In this investigation, ten ICU-specific case scenarios, which included several clinical barriers for mobilization (e.g. mechanical ventilation with an endotracheal tube, delirium or continuous renal replacement therapy), were assessed by different professions of the critical care team. This study aimed to compare the estimation of mobilization capacity of ICU patients among professions.

## Materials and methods

The assessment was based on a previous evaluation using patient scenarios [23], and was reported in accordance with recommendations for the conduction of online surveys [25]. All participants were informed about the study and its voluntary, anonymous approach, as well as the approximate time required to complete the questionnaire. No incentives were offered. The study was approved by the ethical committee of the Faculty of Medicine of the Technical University of Munich (approval 116/17 from 26.04.2017), which waived the requirement for a written informed consent.

### Design

The survey was designed as an online questionnaire containing 23 pages, 32 questions, and 161 items, resulting in a mean of 5 items per question (see S1 Appendix). The questionnaire included in summary 29 multiple-choice questions, two questions with multiple answers and one question with a ranking scale of 0 to 100. The survey covered 5 sections: a) workplace data (self-reported expert status of participants, the definition of early mobilization according to a German guideline [1], presence of protocols, implementation of protocols from 0 (none) until 100 (fully implemented)), b) explanations of terms used in the patient scenarios (Richmond Agitation Sedation Scale, pain, delirium, strength), c) 10 patient scenarios (Table 1 and S1 Appendix), d) barriers to mobilization (three most important barriers out of 20), and e) socio-demographic data (age, work experience, profession, country).

**Table 1:** Case scenarios characteristics.

Variable / Scenario	1^*a*^	2	3	4	5	6	7	8	9	10
**Running Title**	Awake & stable	Comatose & MV with ETT	Awake & MV with ETT	Delirium & MV with TC	Awake & NIV	CRRT & MV with ETT	Delirium & MV with ETT	Pulmonary unstable & MV with TC	Vasopressors & MV with TC	Awake & SAB
**Consciousness**	+^*c*^	comatose	+	Hypoactive Delirium	+	+	Hyperactive Delirium	(+)	Hypoactive Delirium	SAB. ø ICP
**Breathing**	spontaneous	ETT	ETT	TC	NIV	ETT	ETT	TC	TC	spontaneous
**Strength**	+	-	+	+	+	+	+	+	+	+
**Respiratory stability (FiO2)**^***b***^	0.36	0.4	0.5	0.5	0.6	0.45	0.5	0.8	0.5	0.21
**Hemodynamic stability**	+	-	+	+	+	+	+	+	-	+
**Lines**	+	+	+	+	+	CRRT	+	+	+	+
**Days on ICU**	5	10	4	8	20	16	4	9	6	5

^a^ Scenario 1 is a reference example, representing full mobility. Grey cells represent the scenario’s main characteristic.

^b^ FiO2 values of the scenarios are presented.

^c^ + = condition is given, e.g. patient has full consciousness, full strength, is stable, has all routine lines (central venous line, arterial catheter, stomach tube, bladder tube); (+) = reduced condition, e.g. reduced strength;— = unstable condition, e.g. pulmonary stability requiring 80% oxygen, reduced hemodynamic stability, requiring norepinephrine or dobutamine.

Abbreviations: CRRT Continuous Renal Replacement Therapy, ETT Endotracheal Tube, FiO2 Fraction of Oxygen, ICU Intensive Care Unit, SAB Subarachnoid Bleeding, TC Tracheal Cannula.

Participants were free to move back and forth while answering the questionnaire, but had no access to overall results. The questionnaire was self-developed and tested on 19 critical care team members in a pilot phase, after which only minor revisions were undertaken. The results of the pre-test were not included in this analysis. All authors approved the final version.

### Participants

The call for participants was published and advertised via the German Network for Early Mobilization, as well as other related professional networks in Germany, Austria and Switzerland. In order to maximize participation, two reminders were emailed to all members of the network after 2 and 4 weeks. The assessment was administered with SurveyMonkey.

### Case scenarios

We developed ten patient scenarios, including different levels of consciousness and responsiveness [11, 16], different airway devices or level of ventilation support (endotracheal intubation, tracheostomy cannula [26], spontaneous breathing, non-invasive or mechanical ventilation [27]), as well as organ support, such as continuous renal replacement therapy [16], vasopressors [28] or high fraction of oxygen [26] (Table 1 and S1 Appendix). All patient scenarios indicated that this was not to be the first mobilization attempt, as first mobilizations usually require a greater level of caution and flexibility while determining the degree of mobilization that a patient can tolerate [29].

### Outcome measures

The primary outcome for each patient scenario was the ICU Mobility Scale (IMS), from 0 to 10: 0 = none (lying in bed), 1 = sitting in bed, exercises in bed, 2 = passively moved to chair (no standing), 3 = sitting on the edge of the bed, 4 = standing, 5 = transferring from bed to chair, 6 = marching in place (at bedside), 7 = walking with the assistance of 2 or more people, 8 walking with the assistance of 1 person, 9 = walking independently with gait aid, 10 = walking independently without a gait aid [30]. Participants were asked to estimate the mobilization capacity on the ICU Mobility Scale for each patient scenario in realistic conditions, and to estimate the number of required ICU staff necessary to carry it out.

### Data analysis

Data was analyzed with SPSS 22.0 (IBM Corp; New York, NY, USA), and missing data was treated as such. Minimal requirements for inclusion in the analysis were professional details and completion of at least one patient scenario. Nominal and categorical data are reported as numbers and percentages. Due to the non-normal distribution of metrical data, these are reported as median and interquartile range (IQR). Hypotheses were calculated using Kruskal Wallis, based on a double-sided alpha = 0.05. Wilcoxon test was used for a posthoc analysis, with p-values adjusted using the Bonferroni method. A linear mixed-effects model using R Version 3.5.1 (Vienna, Austria) was used for the calculation, using all scenarios and their categorization with the outcome of mobility. Factors regarding scenarios (consciousness, breathing, strength, respiratory stability, hemodynamic stability, lines as described in Table 1) and ICUs (availability of protocols) were categorized and combined with age, work experience and geographical area of the participants. As each participant answered ten scenarios, participants were used as a random factor in the model.

## Results

### Participants

The scenarios were answered by 788 participants, whereas 65% (*n* = 515) completed the minimum requirements. Participants answered all ten scenarios in 92% (*n* = 475), nine scenarios in 7% (*n* = 35) and eight scenarios in 1% (*n* = 5) of cases.

The majority of the participants (72% (*n* = 370)) were nurses, 77% (*n* = 395) of participants worked in Germany, 39% (*n* = 202) were aged between 35–48 years and 26% (*n* = 135) had 5–10 years of work experience (Table 2). Only 34.8% (*n* = 176) of participants could answer the question regarding the definition of early mobilization in accordance to the German guideline correctly. Physical therapists perceived themselves as experts significantly more often than other professions (Table 3). However, only 37% (n = 89) of the self-proclaimed experts answered the question for the precise definition of early mobilization correctly. The median implementation of protocols was 68 [IQR 48–80].

**Table 2:** Participants’ data.

Items	Number (%)
**Profession**	
Nurses	370 (72)
Physical therapists	83 (16)
Physicians	48 (9)
Other specialists^*a*^	14 (3)
**Socio-demographic data**	
Germany	395 (77)
Austria	38 (7)
Switzerland	78 (15)
Other^*b*^	4 (1)
**Age (years)**	
< 35	196 (38)
35–48	202 (39)
49–67	117 (23)
**Job experience (years)**	
In education/study	1 (0)
< 1	17 (3)
1–4	99 (19)
5–10	135 (26)
> 10	131 (25)
> 20	132 (26)
**Professionalism**	
Self-perceived expert status	242 (47)
Answered definition of early mobilization correctly	176 (35)
Experts, who answered definition of early mobilization correctly	89 (37)
**Protocols, implemented on own ICU**	
Analgesia	432 (63)
Sedation	344 (67)
Delirium	267 (52)
Weaning	345 (67)
Early mobilization	162 (32)
Daily inter-professional goals	193 (38)
Automatic order for mobilization	110 (21)
Estimated percentage of existing protocols’ implementation. from 0 = no implementation to 100 = full implementation (median [IQR)]	68 [48–80]

^a^ Respiratory therapists, speech and swallow therapists, occupational therapists

^b^ Luxembourg (n = 2), and missing (n = 2)

**Table 3:** Self-perceived expert status per profession.

Profession	Nurses (n = 367)	Physical therapists^a^ (n = 83)	Physicians (n = 47)	Other specialists^b^ (n = 14)	p-value
Self-perceived expert status	155 (42)	63 (76)	17 (36)	7 (50)	<0.001
Answered definition of early mobilization correctly	131 (36)	26 (31)	15 (32)	6 (43)	0.75
Experts, who answered definition of early mobilization correctly	61 (39)	17 (27)	7 (41)	4 (57)	0.57

^a^ Physical Therapists perceived themselves as experts significantly more often, compared to nurses and physicians (both p<0.001), but not other specialists (p = 0.057).

^b^ Respiratory therapists, speech and swallow therapists, occupational therapists.

### Scenario 1 –awake & stable

**Fig 1:**
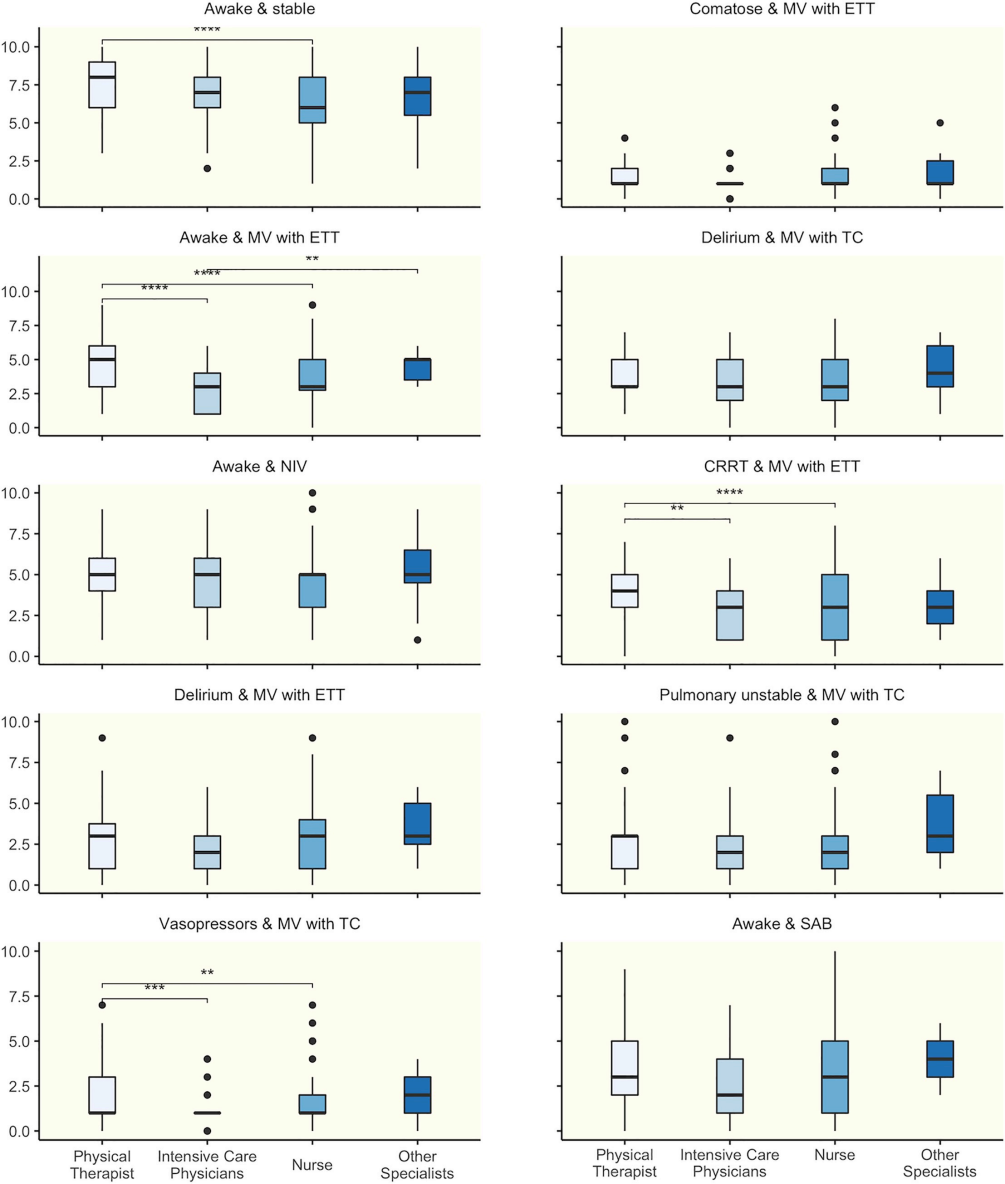
Mobility capacity of ten clinical scenarios scored by different professions. Mobility capacity scored by different professions using the ICU Mobility scale. * p ≤ 0.05. ** p ≤ 0.01. *** p ≤ 0.001. **** p ≤ 0.0001.

The selected mobilization level was 6 [5–8], representing marching in place, with significant differences between nurses and physical therapists (*p*<0.001. Fig 1). Interestingly, 9% would let the patient walk independently, without assistance or gait aid. The median number of health care professionals necessary for mobilization at this level was 2 [1–3].

### Scenario 2 –comatose & mechanical ventilation via endotracheal tube

In this scenario, the median estimated mobilization level was 1 [1, 2], representing active mobilization in bed, without any significant differences among professions in the posthoc tests (Fig 1). Overall, 14% would mobilize the unresponsive and ventilated patient to the edge of the bed. The median number of health care professionals required for mobilization in this level was 2 [1–3], with a lower value of one person in the physician and physical therapist subgroups (*p* = 0.007, see S1 Appendix).

### Scenario 3 –awake & mechanical ventilation via endotracheal tube

In the third scenario, with an awake intubated patient, the median proposed mobilization level was 3 [3–5], which is sitting on the edge of the bed. There are significant differences between physical therapists compared to physicians or nurses (*p*<0.001 for both), as well as physicians and other specialists (*p* = 0.007), with physical therapists and other specialists aiming for higher mobility levels (Fig 1).

Overall, 5% would even attempt to walk with the patient (level 7 or higher), although none of the physicians or other specialists, and only 3% of nurses would try it compared to 14% of physical therapists. On the other hand, physical therapists are the subgroup with the highest percentage (40%) of passive approaches (maximum mobilization level of 2, passively in a chair) compared to 33% in physicians, 25% in nurses and none in other specialists. The median number of required health care professionals was 2 [2–4] (see S1 Appendix).

### Scenario 4 –delirium & mechanical ventilation with a tracheostomy cannula

Median mobilization level in this scenario was 3 [3–5], which is sitting on the edge of a bed, yielding no significant differences among professions (Fig 1). Only a small group of people would try to walk with such a patient (4%). A median number of health care professionals required for mobilization was 2 [2–4], here with significant differences among professions (*p* = 0.012, see S1 Appendix).

### Scenario 5 –awake & non-invasive ventilation

The estimated level of mobilization was 5 [3–6], which represents an active transfer from a bed to a chair, without significant differences in the posthoc comparisons among professions (Fig 1). 12% would try to walk with the patient, with a higher percentage in the subgroup physical therapists (19%) and other specialists (27%), compared to physicians (13%) and nurses (10%). The median number of health care workers required for mobilization was 2 [2–4].

### Scenario 6 –continuous renal replacement therapy & mechanical ventilation via endotracheal tube

In a patient with continuous renal replacement therapy, the estimated level of mobilization was 3 [1–5], corresponding to sitting on the edge of a bed (Fig 1). Physical therapists scored significantly higher compared to nurses (*p*<0.001) and physicians (*p* = 0.0119). Overall, a median of 2.5 [2–4] staff members were estimated to be required for mobilization (see S1 Appendix).

### Scenario 7 –delirium & mechanical ventilation via endotracheal tube

The estimated level of mobilization was 3 [1–4], corresponding to sitting on the edge of bed. There were no significant differences overall (Fig 1). Respondents estimated that 2 [1.25–4] team members would be required to mobilize this patient, with the subgroup nurses and other therapists opting for more staff members (*p* = 0.012, see S1 Appendix).

### Scenario 8 –pulmonary unstable & MV mechanical ventilation via tracheostomy cannula

The median estimated mobilization level was 3 [1–3], which means sitting on the edge of a bed without differences among professions (Fig 1). The median number of health care professionals needed to mobilize that patient was estimated to be 2 [2–4].

### Scenario 9 –vasopressors & MV mechanical ventilation via tracheostomy cannula

In this scenario, the estimated mobilization level was 1 [1–3], which represents active mobilization in the bed (Fig 1). Physical therapists scored significantly higher than nurses (*p* = 0.0164) and physicians (*p*<0.001). However, 22% would attempt to mobilize the patient on level 3 (edge of a bed), especially other specialists (40%) and physical therapists (33%). The median number of health care professionals required for mobilization was 2 [1–3], with physicians only needing one in accordance with their proposed lower mobilization level, while other specialists required a median of four staff members (*p* = 0.020, see S1 Appendix).

### Scenario 10 –awake & subarachnoid hemorrhage

This scenario of neurocritical care resulted in an estimated mobilization level of 3 [1–5], which means sitting on the edge of a bed. 21% of physicians opted for no mobilization at all, and even fewer from other subgroups (12% nurses, 8% physical therapists and 0% other specialists, Fig 1). Again, respondents estimated 2 [1–3] team members as required to mobilize this patient, except physicians who voted for one person (*p* = 0.028, see S1 Appendix).

### Overall model and influencing factors

Profession (*p* = 0.001), expert status (*p* = 0.049), and the knowledge of the definition of early mobilization according to the German guideline (*p* = 0.002) had a significant effect in the linear mixed-effects model over all scenarios adjusted for scenario factors (consciousness, breathing, strength, respiratory stability, hemodynamic stability, lines as described in Table 1), ICU factors (existence of protocols) as well as other respondent characteristics (age, work experience, geographical area, Table 4 and S1 Appendix). Physicians (*p* = 0.001) and nurses (*p* = 0.002) estimated a lower mobilization level compared to physiotherapists, while other specialists did not (*p* = 0.81).

**Table 4:** Linear mixed model over all scenarios.

Factor^a^	Value	95% CI	p-value
Profession compared to Physical therapists			
Physician	-0.7	(-1.1 to -0.3)	0.001
Nurses	-0.4	(-0.7 to -0.2)	0.002
Other specialists	0.1	(-0.5 to 0.6)	0.808
Knowledge of S2e guideline definition	0.3	(0.1 to 0.5)	0.002
Expert	0.2	(0.0 to 0.4)	0.049

^**a**^Linear mixed model with all scenario describing factors (consciousness, breathing, strength, respiratory stability, hemodynamic stability, lines as described in Table 1) being significant (*p* < 0.001). Other factors, such as age, work experience or geographical area of the participants, as well as existing of protocols at the ICU were not significant in the model.

### Barriers

The top three barriers in this survey were hemodynamic and/or pulmonary instability (*n* = 350; 67%), lack of nurses (*n* = 271; 53%), and deep sedation (*n* = 228; 44%) (Table 5). There were no significant differences regarding barriers among professions (see S1 Appendix).

**Table 5:** Top three barriers to mobilization, according to the profession.

Rank of barriers within a profession^a^	Physicians (n = 48)	Nurses (n = 370)	Physical therapists (n = 83)	Others specialists^b^ (n = 14)	Total (n = 515)
TOP 1	Hemodynamic / pulmonary instability 30 (63%)	Hemodynamic / pulmonary instability 252 (68%)	Hemodynamic / pulmonary instability 62 (75%)	Deep sedation 8 (57%)	Hemodynamic / pulmonary instability 350 (68%)
TOP 2	Deep sedation 20 (42%)	Lack of nurses 226 (61%)	Deep sedation 53 (64%)	Hemodynamic / pulmonary instability 6 (43%)	Lack of nurses 271 (53%)
TOP 3	Lack of nurses 19 (40%)	Deep sedation 147 (40%)	Lack of nurses 20 (24%)	Lack of nurses 6 (43%)	Deep sedation 228 (44%)

^a^ Participants were asked to select the three most important barriers

^b^ Includes respiratory therapists, speech and swallow therapists, occupational therapists

## Discussion

We analyzed the assessments of more than 500 critical care team members, who estimated the mobilization capacity of ten different patient scenarios in the ICU. While differences among the professions where found in four scenarios (awake & stable; awake & mechanical ventilation via endotracheal tube; continuous renal replacement therapy & mechanical ventilation via endotracheal tube; vasopressors & mechanical ventilation via tracheostomy cannula), agreement about the possible level of mobilization was observed in six scenarios. Furthermore, ICU staff who knew how early mobilization is defined in the current German guideline suggested a higher target level for the mobilization treatment. In general, physical therapists and other specialists aimed for higher levels of mobilization compared to physicians and nurses, independent of age, job experience, presence of protocols or geographical area. Most cited barriers were patient instability, lack of nurses and deep sedation.

### Mobility levels

No significant differences in estimated mobilization levels among health care professionals were present in scenarios with coma, with delirium, with non-invasive ventilation, with pulmonary instability or with a subarachnoid hemorrhage. For impaired consciousness [31] and delirium [5, 12] evidence of benefits of early mobilization is available in the literature. Early mobilization is typically recommended during non-invasive ventilation [32, 33] and mechanical ventilation [5, 12] while for subarachnoid hemorrhage the evidence in critically ill patients is scarce [13]. However, no simple model or pattern can be derived which scenarios had an interprofessional agreement, so that further investigation is necessary. In accordance with our overall results and the majority of scenarios, however, previous studies also reported differences in the mobilization level of patients according to different professions [16, 34]. These differences can partly be explained by differing education and training [34]. Safety concerns limiting mobilization are common among nurses, as they tend to feel responsible for the integrity of the devices, in particular for the endotracheal tube and the catheters for the renal replacement therapy [35]. Interestingly, Fontela et al. [21] and Jolley et al. [18] found no knowledge differences between physical therapists and nurses according to mobilization. Local standards of education may influence interprofessional differences in knowledge and possibly performance. In addition, there might be distinct ICUs with high levels of mobility across all professions [36]. Another explanation for different mobilization targets might lie on different roles and attitudes [37], leading to varying emphases on specific aspects of mobility. For instance, if a physical therapist focuses on strength and functionality, marching in place might be prioritized [38]. If a nurse focuses on a patient’s well-being, sitting in a chair together with family may be seen as sufficient [39]. Physicians may be more prone to have weaning as a target [26]. Beside these psychosocial and cultural aspects, other facets, such as working habits, inter-professional communication, staff-to-patient ratios, and equipment might contribute to the diverse decision-making [23, 29, 40].

### Staff resources

Estimation of the number of staff necessary for mobilization in each scenario differed in a few cases among professions. The number may depend on the targeted mobility level, and more devices may require more staff [41]. In fact, there is no evidence for a commonly agreed number of staff members, so that it varies according to patient’s condition, targeted mobility level, a mix of professions, and other factors, such as first vs. following mobilization, safety, quality, intensity, duration, and equipment. Guidelines recommend at least two, or even three team members for the mobilization of critically ill patients, but note that the actual number depends on the specific situation [1]. In some of our scenarios, the perception of how much staff is required differed among professions, and this different perception should be acknowledged to improve communication within the team. It must be highlighted that only 32% of the participants used a protocol for early mobilization. This is less than reported in previous studies in Germany [27], but slightly more than in other surveys from the United States, France, or United Kingdom [42]. The presence of mobilization protocols has an increasing impact on the mobilization level of patients [43, 44], but their influence on the individual decisions of health care professionals remains unknown.

### Barriers

In agreement with other reports, we identified patient instability, deep sedation and lack of nurses as the top three barriers against advanced mobilization targets [15, 29]. In contrast to some reports [16, 18, 21, 34], however, there was a similar perception regarding mobilization barriers in our scenarios among the surveyed professions. Garzon-Serrano [16] reported that physical therapists indicate hemodynamic instability as an obstacle to mobilization, but not renal replacement procedures. Berney et al. [34] and Fontela et al. [21] reported similar results, with physicians and nurses rating ventilation status as decisive when setting mobilization targets, while physical therapists perceived sedation state as critical for the decision. Berney explains these differences with varying responsibilities in the ICU team. Lack of nurses is an often-cited barrier, but nurses may oversee possibilities for mobilization [39]. Staff shortage and limited time resources, coupled with the broader responsibilities of caregivers, may favor the higher degree of patient mobilization by physical therapists. Physical therapists are valuable and efficient in terms of mobilization, especially by focusing on the muscular and neurological status of the patient [16]. Nickels et al. [24] reported no differences among professions when conducting a survey in a single ICU with a homogenous culture.

Strategies to overcome these barriers can encompass an inter-professional approach, which has been shown to improve early mobilization [7, 11, 14, 40]. Another important aspect is the use of protocols [45, 46], which can contribute to breaking down the structural barriers. Regular inter-professional meetings as well as inter-professional exchange increase the probability of mobilization [12, 15].

### Strengths and limitations

A strength of this survey is the high number of participating critical care team members from different professions, lowering the risk of a selection bias. The results are limited to voluntary participating respondents with limited generalizability, leading to a possible higher estimation of mobility levels. Another limitation is that the ten scenarios have not been validated. Furthermore, decisions in clinical practice can deviate significantly from fictitious cases. Although only 9% of respondents were physicians, and a higher participation rate might have influenced the results, physicians represent the smallest group within the multi-professional critical care team and early mobilization networks [47]. In addition, not all possible clinical scenarios (e.g. the combination of hemodynamic instability and continuous renal replacement therapy) could be assessed.

## Conclusion

In summary, professions assess the capacity for mobilizing critical care patients differently. A reduced estimation of a patient’s mobility capacity may have an impact on rehabilitation and may limit mobilization. The exact knowledge of guidelines independently leads to a higher target level of mobilization. The key to successful therapy of the intensive care patient is teamwork. To optimally utilize and execute mobilization, an interprofessional understanding of rehabilitation practices and goals is essential. Hence, all professions of the ICU may benefit from interprofessional education, interprofessional rounds and participation in rehabilitation activities.

## Supporting information

S1 Appendix Assessment of mobilization capacity in 10 different ICU scenarios by different professions. PDF with the original (German) survey, detail description of the scenarios, additional tables (required staff of mobilization, the ANOVA calculation of the mixed model and barriers to mobilization).
(PDF)
