# Correction: Assessment of mobilization capacity in 10 different ICU scenarios by different professions

**DOI:** 10.1371/journal.pone.0244890

**Published:** 2020-12-29

**Authors:** The PLOS ONE Staff

There are errors in the author affiliations. The publisher apologizes for the error. The correct affiliations are as follows:

Carsten Hermes^1^ Peter Nydahl^2^, Manfred Blobner^3^, Rolf Dubb^4^, Silke Filipovic^5^, Arnold Kaltwasser^6^, Bernhard Ulm^3^, Stefan J. Schaller^3,7^

**1** CCRN, Bonn, Germany, **2** Nursing Research, Department of Anesthesiology and Intensive Care Medicine, University Hospital of Schleswig-Holstein, Kiel, Germany, **3** Department of Anesthesiology and Intensive Care, Klinikum rechts der Isar, School of Medicine, Technical University of Munich, Munich, Germany, **4** Academy of District Clinics Reutlingen, Reutlingen, Germany, **5** Department of Physiotherapy, University Hospital of Giessen and Marburg, Marburg, Germany, **6** Academy of District Clinics Reutlingen, Reutlingen, Germany, **7** Department of Anesthesiology and Operative Intensive Care Medicine, Charité –Universitätsmedizin Berlin, Berlin, Germany

There are duplicates in the references section. References 7 refers to the same source as reference 12, and reference 8 refers to the same source as reference 31.
