# Supplementary material for: Assessment of mobilization capacity in 10 different ICU scenarios by different professions
Source: PLoS One. 2020 Oct 15;15(10):e0239853. doi: 10.1371/journal.pone.0239853 (PMC7561080; doi:10.1371/journal.pone.0239853)
Supplement: S1 Appendix — PDF with the original (German) survey, detail description of the scenarios, additional tables (required staff of mobilization, the ANOVA calculation of the mixed model and barriers to mobilization). (PDF) [file pone.0239853.s001.pdf]

Appendix - Assessment of mobilization capacity in 10 different ICU scenarios by different professions

APPENDIX 1 – ORIGINAL SURVEY 2

APPENDIX 2 – DESCRIPTION OF THE SCENARIOS 26

SCENARIO 1 - AWAKE & STABLE 26
SCENARIO 2 - COMATOSE & MV WITH ETT 26
SCENARIO 3 - AWAKE & MV WITH ETT 27
SCENARIO 4 - DELIRIUM & MV WITH TC 27
SCENARIO 5 - AWAKE & NIV 28
SCENARIO 6 - CRRT & MV WITH ETT 28
SCENARIO 7 - DELIRIUM & MV WITH ETT 29
SCENARIO 8 - PULMONARY UNSTABLE & MV WITH TC 29
SCENARIO 9 - VASOPRESSORS & MV WITH TC 30
SCENARIO 10 - AWAKE & SAB 30

APPENDIX 3 - REQUIRED STAFF FOR MOBILIZATION 31

APPENDIX 4 - MIXED MODEL – ANOVA CALCULATION 32

APPENDIX 5 - BARRIERS 33

Appendix 1 – Original survey

Liebe Kolleginnen und Kollegen
Das deutsche Netzwerk Frühmobilisierung führt diese Umfrage vom 1.-30. Juli 2017 durch (die Umfragedauer wurde am 30.6. verlängert). Wir fragen nach der Mobilisierbarkeit von Intensivpatienten. Die Umfrage ist in vier Teile gegliedert.

Teil 1 beinhaltet Daten zu Ihrer Intensivstation.

Teil 2 umfasst 10 verschiedene Patientenszenarien, bei der eingeschätzt werden soll:
a) wie weit die jeweiligen Patienten mobilisiert werden könnten und
b) wie viele Mitarbeiter dazu benötigt werden.
In jedem Szenario weist der Titel auf die besondere Situation hin, es werden auch detaillierte Angaben gemacht.

Teil 3 beinhaltet kurz die häufigsten Barrieren in der Mobilisierbarkeit von Intensivpatienten

Teil 4 beinhaltet soziodemographische Daten wie Berufsgruppe usw.

Hinweis: Die Umfrage dauert ca. 10-20 Minuten. Ihre Teilnahme erfolgt freiwillig. Die erhobenen Daten werden anonymisiert gespeichert. Die Umfrage wurde von der Ethikkommission der Medizinischen Fakultät der Technischen Universität München begutachtet (161/17 S). Die Ethikkommission erhob keine Einwände gegen die Studie. Mit Ihrer Teilnahme erklären Sie sich mit den Bedingungen einverstanden.

Vorab bedanken wir uns für Ihre und Eure Teilnahme.

Meine Station

1. Ich selbst halte mich ...

- ◯ für eine/n Experten/Expertin in Frühmobilisierung
- ◯ nicht für eine/n Experten/Expertin in Frühmobilisierung

2. Es gibt auf meiner Station Protokolle, bzw. schriftliche Vereinbarungen zu (Mehrfachantwort)

- ☐ Analgesie
- ☐ Sedierung
- ☐ Delirmanagement
- ☐ Weaning
- ☐ Frühmobilisierung
- ☐ tägliche, interprofessionelle Tagesziele
- ☐ automatische Anordnung zur Frühmobilisierung

3. Ich schätze die bei uns existierenden Protokolle werden in folgendem Umfang umgesetzt (%-Angabe):

0 ———————————————— 100 ☐

Leitlinie

4. Frühmobilisierung gem. DGAI "S2e-Leitlinie: Lagerungstherapie und Frühmobilisation zur Prophylaxe oder Therapie von pulmonalen Funktionsstörungen" ist definiert als Mobilisierung

- ◯ ab Intensivaufnahme
- ◯ innerhalb 72h nach Intensivaufnahme
- ◯ innerhalb einer Woche nach Intensivaufnahme
- ◯ sobald der Patient stabil (z.B. katecholaminfrei) ist

Hintergrundinformation

In den folgenden Szenarios werden Patienten unterschiedlich beschrieben. Damit Sie wissen, worum es geht, sind hier die Definitionen nochmal gelistet

Bewusstsein
Richmond Agitation Sedation Scale:

RASS+ 4
Sehr streitlustig: Offene Streitlust, gewalttätig, unmittelbare Gefahr für das Personal

RASS + 3
Sehr agitiert: Zieht oder entfernt Schläuche oder Katheter; aggressiv

RASS + 2
Agitiert: Häufige ungezielte Bewegung, atmet gegen das Beatmungsgerät

RASS + 1
Unruhig: Ängstlich aber Bewegungen nicht aggressiv oder lebhaft

RASS 0
Aufmerksam ruhig

RASS -1
Schläfrig: Nicht ganz aufmerksam, aber erwacht anhaltend durch Stimme (>10s)

RASS -2
Leichte Sedierung: Erwacht kurz mit Augenkontakt durch Stimme (<10s)

RASS -3
Mäßige Sedierung: Bewegung oder Augenöffnung durch Stimme (aber keinen Augenkontakt)

RASS -4
Tiefe Sedierung: ø Reaktion auf Stimme, aber Bewegung o. Augenöffnung durch körperlichen Reiz

RASS -5
Nicht erweckbar: Keine Reaktion auf Stimme oder körperlichen Reiz

Schmerzen
Es stehen verschiedene Skalen zur Verfügung: hier erfolgt die Einteilung in

- **Keine Schmerzen**
- **Leichte Schmerzen**
- **Mittlere Schmerzen**
- **Starke Schmerzen**

Delir
Ein Delir / Verwirrtheit kann mit der Confusion Assessment Method for the Intensive Care Unit

(CAM-ICU) festgestellt werden

- **CAM-ICU positiv = Delir vorhanden**
- **CAM-ICU negativ = Delir nicht vorhanden**

. **CAM-ICU nicht beurteilbar (z.B. RASS -4/-5)**

Ein Delir kann weiterhin hyperaktiv sein (unruhiger Patient), aber auch hypoaktiv (ruhig, wirkt in sich gekehrt)

Beweglichkeit: Kraftgradeinteilung

- **0/5: keine muskuläre Aktivität, komplette Lähmung**
- **1/5: sichtbare und/oder tastbare Kontraktion ohne Bewegung**
- **2/5: Bewegung unter Ausschaltung der Schwerkraft möglich**
- **3/5: Bewegung gegen die Schwerkraft gerade noch möglich**
- **4/5: Bewegung gegen leichten Widerstand möglich**
- **5/5: Normale Kraft**

Hinweis

Alle Patienten wurden im Verlauf bereits mindestens einmal mobilisiert, sei es passiv im Bett oder bis hin zu aktiver Mobilisierung aus dem Bett.

Szenario 1: Wache, spontan atmende, stabile Patientin

Weibliche Patientin, 58 Jahre alt, Lungenversagen, 5. Tag.
Bewusstsein: aufmerksam ruhig (RASS 0)
Delir: kein Delir (CAM-ICU negativ)
Schmerz: kaum vorhanden
Bewegung: voll beweglich, normale Kraft (Kraftgrad 5/5)
Hämodynamik: Hyperton, "stabil"
Pulmonal: atmet spontan, 4l O2, O2-Sättigung 97%
Zu-/Ableitungen: Sauerstoffbrille, ZVK, Arterie, DK, Ernährungssonde

5. Realistisch würde ich versuchen diese Patientin bis Stufe ... (auswählen) zu mobilisieren

6. Ich benötige dazu ... (Anzahl der jeweiligen Mitarbeiter, inkl. der eigenen Person). Hinweis: Alle ihre Antworten werden zur Gesamtsumme addiert für die Anzahl die Sie für notwendig halten (bei all diesen Fragen).

	0	1	2	3
Ärzte	○	○	○	○
Pflegende oder Physiotherapeuten	○	○	○	○
Physiotherapeuten	○	○	○	○
Pflegende	○	○	○	○

Sonstiges (bitte angeben)

Szenario 2: endotracheal intubierter, kontrolliert beatmeter, komatöser Patient

Männlicher Patient, 74 Jahre alt, Schlaganfall nach OP an Bauchaortenaneurysma, 10. Tag.
Bewusstsein: nicht erweckbar (RASS -5)
Delir: nicht beurteilbar
Schmerz: Schmerz nicht beurteilbar
Bewegung: ohne Tonus, keine muskuläre Aktivität, komplette Lähmung (Kraftgrad 0/5)
Hämodynamik: leicht instabil (Noradrenalin 0,07 µg/kg/min = ca. 5µg/min)
Pulmonal: beatmet, stabil, pO2/FiO2=270 bei FiO2 0.4, Sauerstoffsättigung 98%
Zu-/Ableitungen: endotrachealer Tubus, ZVK, Arterie, DK, Ernährungssonde

7. Realistisch würde ich versuchen diese Patientin bis Stufe ... (auswählen) zu mobilisieren

- ○ 0 Keine Mobilisierung
- ○ 1 Im Bett sitzen, bzw. Übungen
- ○ 2 Passiv in einen Stuhl bewegen
- ○ 3 Auf der Bettkante sitzen
- ○ 4 Stehen vor dem Bett
- ○ 5 Aktiver Transfer in einen Stuhl
- ○ 6 Auf der Stelle gehen (zwei Schritte jedes Bein)
- ○ 7 Gehen mit ≥ 2 Personen > 5m
- ○ 8 Gehen mit 1 Person > 5m
- ○ 9 Gehen mit Hilfsmittel > 5m
- ○ 10 Unabhängiges Gehen

8. Ich benötige dazu ... (Anzahl der jeweiligen Mitarbeiter)

	0	1	2	3
Ärzte	○	○	○	○
Pflegende oder Physiotherapeuten	○	○	○	○
Physiotherapeuten	○	○	○	○
Pflegende	○	○	○	○

Sonstiges (bitte angeben)

Szenario 3: wacher, endotracheal intubierter, beatmeter Patient

Männlicher Patient, 68 Jahre alt, Aspirationspneumonie, Tag 4
Gewicht & Größe: "normal"
Bewusstsein: aufmerksam ruhig (RASS 0)
Delir: nicht vorhanden (CAM-ICU negativ)
Schmerz: schmerzfrei
Bewegung: leichte Einschränkung, Bewegung gegen leichten Widerstand möglich (Kraftgrad 4/5)
Hämodynamik: "stabil"
Pulmonal: beatmet, stabil, pO2/FiO2=180 bei FiO2 0.5, Sauerstoffsättigung 97%
Zu-/Ableitungen: endotrachealer Tubus, ZVK, Arterie, DK, Ernährungssonde

9. Realistisch würde ich versuchen diese Patientin bis Stufe ... (auswählen) zu mobilisieren

10. Ich benötige dazu ... (Anzahl der jeweiligen Mitarbeiter)

	0	1	2	3
Ärzte	◯	◯	◯	◯
Pflegende oder Physiotherapeuten	◯	◯	◯	◯
Physiotherapeuten	◯	◯	◯	◯
Pflegende	◯	◯	◯	◯

Sonstiges (bitte angeben)

Szenario 4: tracheostomierte, assistiert beatmete Patientin, eingeschränkt mobil

Weibliche Patientin, 53 Jahre alt, Z.n. Sepsis, 8. Tag
Bewusstsein: schläfrig (RASS -1)
Delir: vorhanden (CAM-ICU positiv: hypoaktiv)
Schmerz: schmerzfrei
Bewegung: leichte Einschränkung, Bewegung gegen leichten Widerstand möglich (Kraftgrad 4/5)
Hämodynamik: "stabil"
Pulmonal: beatmet, stabil, pO2/FiO2=170 bei FiO2 0.5, Sauerstoffsättigung 97%
Zu-/Ableitungen: Trachealkanüle, ZVK, Arterie, DK, Ernährungssonde

11. Realistisch würde ich versuchen diese Patientin bis Stufe ... (auswählen) zu mobilisieren

12. Ich benötige dazu ... (Anzahl der jeweiligen Mitarbeiter)

	0	1	2	3
Ärzte	◯	◯	◯	◯
Pflegende oder Physiotherapeuten	◯	◯	◯	◯
Physiotherapeuten	◯	◯	◯	◯
Pflegende	◯	◯	◯	◯

Sonstiges (bitte angeben)

Szenario 5: assistiert beatmete Patientin mit nicht-invasiver Maskenbeatmung

Weibliche Patientin, 72 Jahre alt, exarzerbierte COPD, 20. Tag
Bewusstsein: aufmerksam ruhig (RASS 0)
Delir: nicht vorhanden (CAM-ICU negativ)
Schmerz: schmerzfrei
Bewegung: leichte Einschränkung, Bewegung gegen leichten Widerstand möglich (Kraftgrad 4/5)
Hämodynamik: "stabil"
Pulmonal: beatmet (NIV-Maske), stabil, pO2/FiO2=130 bei FiO2 0.6, Sauerstoffsättigung 94%
Zu-/Ableitungen: NIV, ZVK, Arterie, DK, Ernährungssonde

13. Realistisch würde ich versuchen diese Patientin bis Stufe ... (auswählen) zu mobilisieren

14. Ich benötige dazu ... (Anzahl der jeweiligen Mitarbeiter)

	0	1	2	3
Ärzte	○	○	○	○
Pflegende oder Physiotherapeuten	○	○	○	○
Physiotherapeuten	○	○	○	○
Pflegende	○	○	○	○

Sonstiges (bitte angeben)

Szenario 6: endotracheal intubierter, assistiert beatmeter Patient, laufendes Nierenersatzverfahren

Männlicher Patient, 50 Jahre alt, Pneumonie, 16. Tag
Bewusstsein: aufmerksam ruhig (RASS 0)
Delir: nicht vorhanden (CAM-ICU negativ)
Schmerz: schmerzfrei
Bewegung: Bewegung gegen leichten Widerstand möglich (Kraftgrad 4/5)
Hämodynamik: "stabil"
Pulmonal: beatmet, stabil, pO2/FiO2=230 bei FiO2 0.45, Sauerstoffsättigung 98%
Zu-/Ableitungen: endotrachealer Tubus, laufendes Nierenersatzverfahren (Shaldonkatheter in Subclavia) das nicht zur Mobilisierung diskonnektiert wird, ZVK, Arterie, DK, Ernährungssonde

15. Realistisch würde ich versuchen diese Patientin bis Stufe ... (auswählen) zu mobilisieren

16. Ich benötige dazu ... (Anzahl der jeweiligen Mitarbeiter)

	0	1	2	3
Ärzte	○	○	○	○
Pflegende oder Physiotherapeuten	○	○	○	○
Physiotherapeuten	○	○	○	○
Pflegende	○	○	○	○

Sonstiges (bitte angeben)

Szenario 7: endotracheal intubierte, assistiert beatmete Patientin im hyperaktiven Delir

Weibliche Patientin, 59 Jahre alt, Lungenversagen, 4. Tag
Bewusstsein: agitiert (RASS +2)
Delir: hyperaktives Delir vorhanden (CAM-ICU positiv)
Schmerz: leicht vorhanden
Bewegung: normale Kraft (Kraftgrad 5/5)
Hämodynamik: Hyperton, "stabil"
Pulmonal: beatmet, instabil, pO2/FiO2=170 bei FiO2 0.5, Sauerstoffsättigung 97%
Zu-/Ableitungen: endotrachealer Tubus, ZVK, Arterie, DK, Ernährungssonde

17. Realistisch würde ich versuchen diese Patientin bis Stufe ... (auswählen) zu mobilisieren

18. Ich benötige dazu ... (Anzahl der jeweiligen Mitarbeiter)

	0	1	2	3
Ärzte	○	○	○	○
Pflegende oder Physiotherapeuten	○	○	○	○
Physiotherapeuten	○	○	○	○
Pflegende	○	○	○	○

Sonstiges (bitte angeben)

Szenario 8: tracheostomiert assistiert beatmeter, pulmonal instabiler Patient

Männlicher Patient, 70 Jahre alt, Pneumonie, 9. Tag
Bewusstsein: leichte Sedierung (RASS -2),
Delir: nicht vorhanden (CAM-ICU negativ)
Schmerz: leichte Schmerzen
Bewegung: normale Kraft (Kraftgrad 5/5)
Hämodynamik: "stabil"
Pulmonal: beatmet, stabil, pO_2/FiO_2=130 bei FiO_2 0.8, Sauerstoffsättigung 95%
Zu-/Ableitungen: Tracheostomiert, ZVK, Arterie, DK, Ernährungssonde

19. Realistisch würde ich versuchen diese Patientin bis Stufe ... (auswählen) zu mobilisieren

20. Ich benötige dazu ... (Anzahl der jeweiligen Mitarbeiter)

	0	1	2	3
Ärzte	○	○	○	○
Pflegende oder Physiotherapeuten	○	○	○	○
Physiotherapeuten	○	○	○	○
Pflegende	○	○	○	○

Sonstiges (bitte angeben)

Szenario 9: tracheostomiert assistiert beatmete Patientin mit Katecholaminen

Weibliche Patientin, 61 Jahre alt, Sepsis, 6. Tag

Bewusstsein: schläfrig (RASS -1)
Delir: hypoaktiv vorhanden (CAM-ICU positiv)
Schmerz: schmerzfrei
Bewegung: Bewegung gegen leichten Widerstand möglich (Kraftgrad 4/5)
Hämodynamik: "stabil" bei hohen Dosen Arterenol (0,4 µg/kg/min = ca. 28µg/min), Dobutamin (12 µg/kg/min = ca. 0.8mg/min)
Pulmonal: beatmet, stabil, pO2/FiO2=180 bei FiO2 0.5, Sauerstoffsättigung 97%
Zu-/Ableitungen: Trachealkanüle, ZVK, Arterie, DK, Ernährungssonde

21. Realistisch würde ich versuchen diese Patientin bis Stufe ... (auswählen) zu mobilisieren

22. Ich benötige dazu ... (Anzahl der jeweiligen Mitarbeiter)

	0	1	2	3
Ärzte	◯	◯	◯	◯
Pflegende oder Physiotherapeuten	◯	◯	◯	◯
Physiotherapeuten	◯	◯	◯	◯
Pflegende	◯	◯	◯	◯

Sonstiges (bitte angeben)

Szenario 10: Patientin mit SAB

Weibliche Patientin, 51 Jahre alt, leichte Subarachnoidalblutung (Hunt&Hess 2), Tag 5, keine Hirndrücke, gering erhöhte Flüsse
Bewusstsein: schläfrig (RASS -1)
Delir: Delir nein (CAM-ICU negativ)
Schmerz: Schmerz VAS 3
Bewegung: normale muskuläre Kraft, (Kraftgrad 5/5)
Hämodynamik: "stabil"
Pulmonal: Spontanatmend, Sauerstoffsättigung 98%
Zu-/Ableitungen: ZVK, Arterie, DK, Hirndrucksonde

23. Realistisch würde ich versuchen diese Patientin bis Stufe ... (auswählen) zu mobilisieren

24. Ich benötige dazu ... (Anzahl der jeweiligen Mitarbeiter)

	0	1	2	3
Ärzte	○	○	○	○
Pflegende oder Physiotherapeuten	○	○	○	○
Physiotherapeuten	○	○	○	○
Pflegende	○	○	○	○

Sonstiges (bitte angeben)

Barrieren

Unten ist eine Liste möglicher Barrieren zur Mobilisierung. Bitte klicken Sie die 3 aus Ihrer Sicht wichtigsten Barrieren an.

25. Die 3 wichtigsten Barrieren sind ...

- ☐ Hämodynamische/pulmonale Instabilität
- ☐ Zu- und Ableitungen
- ☐ Zu tiefe Sedierung
- ☐ Hyperaktives Delir
- ☐ Mangel an Pflegepersonal
- ☐ Mangel an Physiotherapeuten
- ☐ Mangel an Therapeuten
- ☐ Mangel an Ärzten
- ☐ Fehlendes Equipment
- ☐ Fehlendes Mobilisierungsprotokoll
- ☐ Zu frühe Verlegung der Patienten
- ☐ Unklare Verantwortungen
- ☐ Kommunikation zwischen den Berufsgruppen
- ☐ Keine interprofessionelle Visiten
- ☐ Keine Tagesziele
- ☐ Fehlende Kultur der Mobilität
- ☐ Sicherheitsbedenken
- ☐ Zu wenig Wissen und Training
- ☐ Zu wenig Unterstützung
- ☐ Unwillen, das Verhalten zu ändern

Soziodemographische Daten

26. Mein Alter

- <35
- 35-48
- 49-67

27. Meine Berufserfahrung auf Intensivstation

- zurzeit noch in Ausbildung/Studium
- < 1 Jahr
- 1-4 Jahre
- 5-10 Jahre
- > 10 Jahre
- > 20 Jahre

28. Ich arbeite als ...

- Atmungstherapeut
- Assistenzarzt in Weiterbildung
- Ergotherapeut
- Facharzt mit abgeschlossener Zusatzweiterbildung Intensivmedizin
- Facharzt ohne abgeschlossener Zusatzweiterbildung Intensivmedizin
- Oberarzt auf einer Intensivstation (>50% auf Intensivstation tätig)
- Chefarzt
- Pflegende
- Pflegende (Fachweiterbildung A&I, 2-jährig)
- Physiotherapeut
- Pflege sonstige
- Sonstiges (bitte angeben)

29. Ich arbeite in

- ◯ Deutschland
- ◯ Österreich
- ◯ Schweiz
- ◯ Luxemburg
- ◯ Sonstiges Land

In welchem Bundesland arbeiten Sie?

30. In welchem Bundesland arbeiten Sie?

- ◯ Baden-Württemberg
- ◯ Bayern
- ◯ Berlin
- ◯ Brandenburg
- ◯ Bremen
- ◯ Hamburg
- ◯ Hessen
- ◯ Mecklenburg-Vorpommern
- ◯ Niedersachsen
- ◯ Nordrhein-Westfalen
- ◯ Rheinland-Pfalz
- ◯ Saarland
- ◯ Sachsen
- ◯ Sachsen-Anhalt
- ◯ Schleswig-Holstein
- ◯ Thüringen

In welchem österreichischem Bundesland arbeiten Sie?

31. In welchem Bundesland arbeiten Sie?

- ◯ Vorarlberg
- ◯ Tirol
- ◯ Salzburg
- ◯ Oberösterreich
- ◯ Kärnten
- ◯ Steiermark
- ◯ Burgenland
- ◯ Niederösterreich
- ◯ Wien

In welchem Kanton arbeiten Sie?

32. In welchem Kanton arbeiten Sie?

- Aargau
- Appenzell Ausserrhoden
- Appenzell Innerrhoden
- Basel-Land
- Basel-Stadt
- Bern
- Fribourg
- Genève
- Glarus
- Graubünden
- Jura
- Luzern
- Neuchâtel
- Nidwalden
- Obwalden
- St.Gallen
- Schaffhausen
- Schwyz
- Solothurn
- Thurgau
- Ticino
- Uri
- Vaud
- Valais
- Zug
- Zürich

Vielen Dank

Wenn Sie bereit sind an Folgebefragungen teilzunehmen, drücken Sie bitte den unten stehenden Link. Dieser öffnet eine von dieser Umfrage unabhängige Mailingliste, wo Sie ihre Emailadresse eintragen können. Damit bleiben Ihre Antworten garantiert anonymisiert. Wir würden uns freuen, wenn Sie bereit wären, sich einzutragen.

Link: https://lists.lrz.de/mailman/listinfo/mob-icu (Es ist ausreichend lediglich die Emailadresse einzugeben und abzuschicken; die Angabe des Namens oder eines von Ihnen gewünschten Passwortes ist freiwillig.)

Sie haben uns mit der Beantwortung dieser Fragen sehr geholfen!
Danke vielmals!

Appendix 2 – Description of the scenarios

Scenario 1 - Awake & stable

Headline	awake, spontaneous breathing, stable patient
Description	female patient, 58 years old with lung failure, 5th day of ICU
Consciousness	attentive, untroubled (RASS 0)
Delirium	no delirium (CAM-ICU negative)
Pain	hardly present
Mobility/Strength	fully mobile, normal strength (MRC 5/5)
Hemodynamics	hypertensive, “stable”
Pulmonary	breathing spontaneously, 4l O2/min, O2-Saturation 97%
Lines	nasal oxygen, central venous catheter, arterial line, urinary catheter, feeding tube

Scenario 2 - Comatose & MV with ETT

Headline	endotracheally intubated, mechanically ventilated, comatose patient
Description	male patient, 74 years old with a stroke after surgery of an abdominal aortic aneurysm, 10th day of ICU
Consciousness	not arousable (RASS -5)
Delirium	not evaluable
Pain	not evaluable
Mobility/Strength	without tonicity, no muscle activity, completely paralysis (MRC 0/5)
Hemodynamics	slightly unstable (norepinephrine 0.07 µg/kg/min = ca. 0.5 µg/min)
Pulmonary	ventilated, stable, pO2/FiO2 = 270 with 0.4 FiO2, O2-Saturation 98%
Lines	endotracheal tube, central venous catheter, arterial line, urinary catheter, feeding tube

Scenario 3 - Awake & MV with ETT

Headline	awake, endotracheally intubated, mechanically ventilated patient
Description	male patient, 68 years old, aspiration pneumonia, 4th day of ICU, normal height & weight
Consciousness	alert & calm (RASS 0)
Delirium	no delirium (CAM-ICU negative)
Pain	without pain
Mobility/Strength	light restriction, mobility against light resistance possible (MRC 4/5)
Hemodynamics	stable
Pulmonary	ventilated, stable, pO2/ FiO2 = 180 with FiO2 0.5, O2-Saturation 97%
Lines	endotracheal tube, central venous catheter, arterial line, urinary catheter, feeding tube

Scenario 4 - Delirium & MV with TC

Headline	tracheotomized, assisted ventilated patient, restricted mobility
Description	female patient, 53 years old, status post sepsis, 8th day of ICU
Consciousness	drowsy (RASS -1)
Delirium	delirium present (CAM-ICU positive, hypoactive)
Pain	painless
Mobility/Strength	light restriction, mobility against light resistance (MRC 4/5)
Hemodynamics	stable
Pulmonary	ventilated, stable, pO2/FiO2 = 170 by FiO2 0.5%, O2-Saturation 97%
Lines	tracheostomy cannula, central venous catheter, arterial line, urinary catheter, feeding tube

Scenario 5 - Awake & NIV

Headline	assistent ventilated patient with non-invasive mask ventilation
Description	female patient, 72 years old, acute exacerbation of COPD, 20th day of ICU
Consciousness	alert & calm (RASS 0)
Delirium	no delirium (CAM-ICU negative)
Pain	painless
Mobility/Strength	light restriction, mobility against light resistance (MRC 4/5)
Hemodynamics	stable
Pulmonary	ventilated (NIV-mask), stable, pO2/FiO2 = 130 with 0.6 FiO2, O2-Saturation 94%
Lines	NIV, central venous catheter, arterial line, urinary catheter, feeding tube

Scenario 6 - CRRT & MV with ETT

Headline	endotracheally intubated, assistant ventilation, with renal replacement therapy
Description	male patient, 50 years old, pneumonia, 16th day of ICU
Consciousness	alert & calm (RASS 0)
Delirium	no delirium (CAM-ICU negative)
Pain	painless
Mobility/Strength	mobility against light resistance possible (MRC 4/5)
Hemodynamics	stable
Pulmonary	ventilated, stable, pO2/FiO2 = 230 with FiO2 0.45, O2-Saturation 98%
Lines	endotracheal tube, running renal replacement therapy (shaldon catheter in the subclavian vein) not disconnected during mobilization, central venous catheter, arterial line, urinary catheter, feeding tube

Scenario 7 - Delirium & MV with ETT

Headline	endotracheally intubated, assistant ventilated patient with hyperactive delirium
Description	female patient, 59 years old, lung failure, 4th day of ICU
Consciousness	agitated (RASS +2)
Delirium	hyperactive delirium (CAM-ICU positive)
Pain	slightly present
Mobility/Strength	normal strength (MRC 5/5)
Hemodynamics	hypertensive, stable
Pulmonary	ventilated, stable, pO2/ FiO2 = 170 by FiO2 0.5%, O2-Saturation 97%
Lines	endotracheal tube, central venous catheter, arterial line, urinary catheter, feeding tube

Scenario 8 - Pulmonary unstable & MV with TC

Headline	tracheotomized, assistant ventilated, pulmonary unstable patient
Description	male patient, 70 years old, pneumonia, 9th day of ICU
Consciousness	light sedation (RASS -2)
Delirium	no delirium, (CAM-ICU negative)
Pain	slightly present
Mobility/Strength	normal strength (MRC 5/5)
Hemodynamics	stable
Pulmonary	ventilated, unstable, pO2/FiO2 = 130 with FiO2 0.8, O2-Saturation 95%
Lines	tracheostomy cannula, central venous catheter, arterial line, urinary catheter, feeding tube

Scenario 9 - Vasopressors & MV with TC

Headline	tracheotomized, assistant ventilated patient with catecholamines
Description	female patient, 61 years old, sepsis, 6th day of ICU
Consciousness	drowsy (RASS -1)
Delirium	hypoactive, present (CAM-ICU positive)
Pain	painless
Mobility/Strength	mobility against light resistance possible (4/5)
Hemodynamics	“stable” with high doses of norepinephrine (0.4 µg/kg/min = approx. 28 µg/min) and dobutamine (12 µg/kg/min = approx. 0.8 mg/min)
Pulmonary	ventilated, stable, pO2/ FiO2 = 180 with FiO2 0.5, O2-Saturation 97%
Lines	tracheostomy cannula, central venous catheter, arterial line, urinary catheter, feeding tube

Scenario 10 - Awake & SAB

Headline	patient with SAB
Description	female patient, 51 years old, light subarachnoidal hemorrhage (Hunt&Hess 2), 5th day of ICU, no intracranial pressure, slightly increased arterial flow
Consciousness	sleepy (RASS -1)
Delirium	no delirium (CAM-ICU negative)
Pain	slightly present (VAS 3)
Mobility/Strength	normal muscular strength (MRC 5/5)
Hemodynamics	stable
Pulmonary	spontaneous breathing, O2-Saturation 98%
Lines	central venous catheter, arterial line, urinary catheter, intracranial pressure probe

Appendix 3 - Required staff for mobilization

Scenario/ Profession	Total[a]	Physicians	Nurses	Physical Therapists	Other Specialists[b]	p-value
1 Awake	2 (1-3)	2 (1-2.75)	2 (1-3)	1.5 (1-2)	2 (1-3)	0.179
2 Coma	2 (1-3)	1 (0-2)	2 (1-3.75)	1 (1-1.75)	1.5 (0.75-2.25)	0.007
3 ETT	2 (2-4)	3 (2-3)	3 (2-4)	2 (2-3)	2 (2-3.5)	0.083
4 TC	2 (2-4)	3 (2-4)	3 (2-4)	2 (2-2)	2 (2-3)	0.012
5 NIV	2 (2-4)	2 (2-3)	2 (2-4)	2 (1-2)	2 (2-3)	0.073
6 CRRT	2.5 (2-4)	3 (1.5-3)	2 (2-4)	3 (2-3)	2 (2-2.75)	0.693
7 Delirium	2 (1.25-4)	2 (1-3)	3 (2-4)	2 (1-2)	3 (2-3)	0.012
8 FiO_2 0.8	2 (2-4)	2 (2-3.5)	3 (2-4)	2 (1-2)	3 (2-4)	0.080
9 Vasopressor	2 (1-3)	1 (0-1.5)	2 (1-3)	2 (1-2.75)	4 (2-4)	0.020
10 SAB	2 (1-3)	1 (0-2)	2 (1-3)	2 (1-2.75)	3 (2-3)	0.028

a Data are reported in Median (Interquartile Range)
b includes Respiratory Therapists, Speech and Swallow Therapists, Occupational Therapists

Abbreviations: CRRT Continuous Renal Replacement Therapy, ETT Endotracheal Tube, FiO2 Fraction of Oxygen, ICU Intensive Care Unit, SAB Sub Arachnoid Bleeding, TC Tracheal Cannula

Appendix 4 - Mixed model – ANOVA calculation

ANOVA over all scenarios with all factors included.

Variable	F-value	p-value	Significance
(Intercept)	50.5	<0.001	***
Profession	5.3	0.001	**
Knowledge S2e guideline	9.7	0.002	**
Expert	3.9	0.049	*
Geographical Area	1.8	0.179	
Age	1.3	0.290	
Work experience	1.0	0.428	
**Protocol for**			
... mobilization	1.0	0.321	
... daily Goals	0.6	0.428	
... sedation	0.3	0.569	
... orders	0.2	0.635	
... delirium	0.1	0.782	
... weaning	0.1	0.804	
... analgesia	0.0	0.893	
**Scenario factor**			
Consciousness	90.3	<0.001	***
Hemodynamic instability	836.4	<0.001	***
Pulmonary instability	178.6	<0.001	***
ETT	297.4	<0.001	***
TC	66.6	<0.001	***
NIV	19.5	<0.001	***
CRRT	61.6	<0.001	***
Neurocritical	1581.2	<0.001	***

* p ≤ 0.05. ** p ≤ 0.01. *** p ≤ 0.001. **** p ≤ 0.0001

Appendix 5 - Barriers

Barriers[a] / Profession (n,%)	Physicians (n=48)	Nurses (n=370)	Physical Therapists (n=78)	Other Specialist[b] (n=15)	Total (n=515)
Hemodynamic / pulmonary instability	30 (62.5)	252 (68.1)	62 (74.7)	6 (42.9)	350 (67.9)
Lack of nurses	19 (39.6)	226 (61.1)	20 (24.1)	6 (42.9)	271 (52.6)
Deep sedation	20 (41.7)	147 (39.7)	53 (63.9)	8 (57.1)	228 (44.3)
Lack of knowledge	11 (22.9)	87 (23.5)	8 (9.6)	2 (14.3)	108 (20.9)
Missing culture	15 (31.3)	73 (19.7)	14 (16.9)	1 (7.1)	103 (20)
Lack of Physical Therapists	12 (25)	76 (20.5)	9 (10.8)	2 (14.3)	99 (19.2)
Unwillingness to change behavior	8 (16.7)	63 (17)	12 (14.5)	3 (21.4)	86 (16.7)
Safety concerns	6 (12.5)	62 (16.8)	14 (16.9)	3 (21.4)	85 (16.5)
Hyperactive delirium	14 (29.2)	45 (12.2)	21 (25.3)	0 (0)	80 (15.5)
Missing equipment	3 (6.3)	49 (13.2)	10 (12)	1 (7.1)	63 (12.2)
Lines and tubes	7 (14.6)	35 (9.5)	7 (8.4)	0 (0)	49 (9.5)
Interprofessional communication	5 (10.4)	17 (4.6)	10 (12)	4 (28.6)	36 (6.9)
Missing protocol	3 (6.3)	25 (6.8)	4 (4.8)	1 (7.1)	33 (6.4)
Missing daily goals	3 (6.3)	26 (7)	3 (3.6)	1 (7.1)	33 (6.4)
Missing support	1 (2.1)	20 (5.4)	6 (7.2)	1 (7.1)	28 (5.4)
Lack of therapists	5 (10.4)	16 (4.3)	1 (1.2)	1 (7.1)	23 (4.5)
Missing interprofessional rounds	2 (4.2)	10 (2.7)	5 (6)	0 (0)	17 (3.3)
Unclear responsibilities	1 (2.1)	6 (1.6)	4 (4.8)	1 (7.1)	12 (2.3)
Early discharge	1 (2.1)	4 (1.1)	1 (1.2)	0 (0)	6 (1.2)
Lack of physicians	0 (0)	3 (0.8)	0 (0)	2 (14.3)	5 (1)

Data are reported in number (percent)
[a] Participants were asked to choose the three most important barriers
[b] includes Respiratory Therapists, Speech and Swallow Therapists, Occupational Therapists
